# Full‐active pharmaceutical ingredient nanosensitizer for augmented photoimmunotherapy by synergistic mitochondria targeting and immunogenic death inducing

**DOI:** 10.1002/mco2.756

**Published:** 2024-11-09

**Authors:** Xianghui Li, Haoran Wang, Zhiyan Li, Song Liu, Yuanyuan Chen, Zhuren Ruan, Zhijian Yao, Gao Wei, Cunwei Cao, Wenjun Zheng, Wenxian Guan

**Affiliations:** ^1^ Department of Dermatology and Venereology The First Affiliated Hospital of Guangxi Medical University Nanning China; ^2^ Department of Gastrointestinal Surgery Affiliated Nanjing Drum Tower Hospital Nanjing University Medical School Nanjing China; ^3^ State Key Laboratory on Technologies for Chinese Medicine Pharmaceutical Process Control and Intelligent Manufacture Nanjing University of Chinese Medicine Nanjing China

**Keywords:** full‐API nanosystem, heat shock protein, immunogenic cell death, mitochondria targeting, photoimmunotherapy

## Abstract

The precise and effective activation of the immune response is crucial in promising therapy curing cancer. Photoimmunotherapy (PIT) is an emerging strategy for precise regulation and highly spatiotemporal selectivity. However, this approach faces a significant challenge due to the off‐target effect and the immunosuppressive microenvironment. To address this challenge, a nanoscale full‐active pharmaceutical ingredient (API) photo‐immune stimulator was developed. This formulation overcomes the limitations of PIT by strengthening the ability to penetrate tumors deeply and inducing precise and potent mitochondria‐targeted dual‐mode photodynamic therapy and photothermal therapy. Along with inhibiting overexpressed Hsp90, this nanosensitizer in turn improves the immunosuppressive microenvironment. Ultimately, this mitochondria‐targeted PIT demonstrated potent antitumor efficacy, achieving a remarkable inhibition rate of ≥95% for both established primary tumors and distant abscopal tumors. In conclusion, this novel self‐delivery full‐API nanosystem enhances the efficacy of phototherapy and reprograms the immunosuppressive microenvironment, thereby holding great promise in the development of precise and effective immunotherapy.

## Graphical abstract

A nanoscale full‐active pharmaceutical ingredient (API) photo‐immune stimulator was developed to overcome the limitations of PIT. Along with strengthening the ability to penetrate tumors deeply and inducing precise and potent mitochondria‐targeted immunotherapy, this photoimmunotherapy demonstrated potent antitumor efficacy, achieving a remarkable inhibition rate >95% for both established primary tumors and distant abscopal tumors.

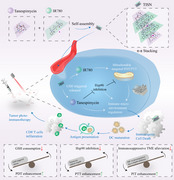

## 1 INTRODUCTION

Immunotherapy has illuminated the horizon of cancer treatment, offering new hope, particularly for patients diagnosed at an advanced stage.^1^, ^2^ Despite the notable advancements in recent years, the therapeutic efficacy of immunotherapy is still impeded by the tumor's immunosuppressive microenvironment and the adverse reactions associated with off‐target effects. These factors continue to be the main hurdles to the broader clinical adoption of this promising treatment modality.^3^, ^4^, ^5^ Moreover, the inclusion of additional drug carriers or stabilizers can introduce unforeseen responses and diminish the potency of the API.^6^, ^7^ The contradiction between robust anticancer immune responses and minimized side effects underscores the pressing need for innovative therapeutic approaches that can precisely and effectively stimulate systemic anticancer immunity.

Photoimmunotherapy (PIT), which leverages the principles of photodynamic therapy (PDT) or photothermal therapy (PTT) to induce an immune response, presents itself as an innovative and promising strategy. Its appeal lies in the spatial and temporal selectivity and the noninvasive nature of phototherapeutic interventions.^8^, ^9^ However, the effectiveness of PIT is often constrained by the inadequate immune response elicited by single‐mode stimulation and the challenging tumor immunosuppressive microenvironment.^10^

Dual‐mode PIT, which integrates PDT and PTT, has demonstrated its potential to stimulate immune responses effectively. PDT effectively generates reactive oxygen species (ROS), which in turn induces oxidative stress within the targeted cells, while PTT triggers endoplasmic reticulum stress through hyperthermia, both of which are viable for PIT applications.^11^, ^12^ Our previous research highlighted that mitochondria‐targeted therapies are a potent strategy for enhancing the effects of PDT and PTT using IR780.^13^, ^14^ Mitochondria, often referred to as the ‘powerhouses of the cell,’ are responsible for the production of adenosine triphosphate (ATP) via oxidative phosphorylation. This vital metabolic pathway that consumes a significant amount of oxygen and is integral to cell proliferation and other biological processes. Mitochondria are also sensitive to both ROS and heat, playing a role in the pathways of apoptosis and necrosis. When mitochondria are compromised, the consumption of oxygen and the production of ATP are drastically curtailed due to the obstruction of the oxidative phosphorylation pathway. Furthermore, oxygen is the precursor to ROS, and ATP is essential for the activation of Heat Shock Protein 90 (Hsp90), both of which could potentially amplify the effects of PDT and PTT, culminating in a robust yet controlled anticancer immune response. However, intracellular reducing substances (e.g., glutathione [GSH]) decrease ROS generation in PDT while resistant mechanisms (e.g., heat‐shock protein90, Hsp90) limit PTT efficacy,^15^, ^16^ and both of their levels are usually upregulated in tumor cells.^17^, ^18^, ^19^, ^20^

Antitumor therapeutic interventions combating resistant mechanisms aroused researchers’ interest.^21^ Tanespimycin, a potent Hsp90 inhibitor, has exhibited a 100‐fold greater binding affinity for Hsp90 proteins derived from tumor cells compared with those from normal cells.^22^ Tanespimycin was evaluated in a series of phase II clinical trials but failed as monotherapy against cancer.^23^, ^24^ Given the individual shortcomings of IR780 and Tanespimycin, the combined strategy may be an optimized solution for precise and effective mitochondria‐targeted PIT (Mt‐PIT) combating tumor‐resistant mechanisms. Moreover, recent research has highlighted the potential of Tanespimycin to induce immunogenic cell death (ICD) when delivered into tumor tissue.^25^ Thus, the amplified PIT via the ICD pathway may be achieved through a well‐designed nanosystem, which triggers a potent anticancer immune response and reverses the suppressive microenvironment.

**FIGURE 1:**
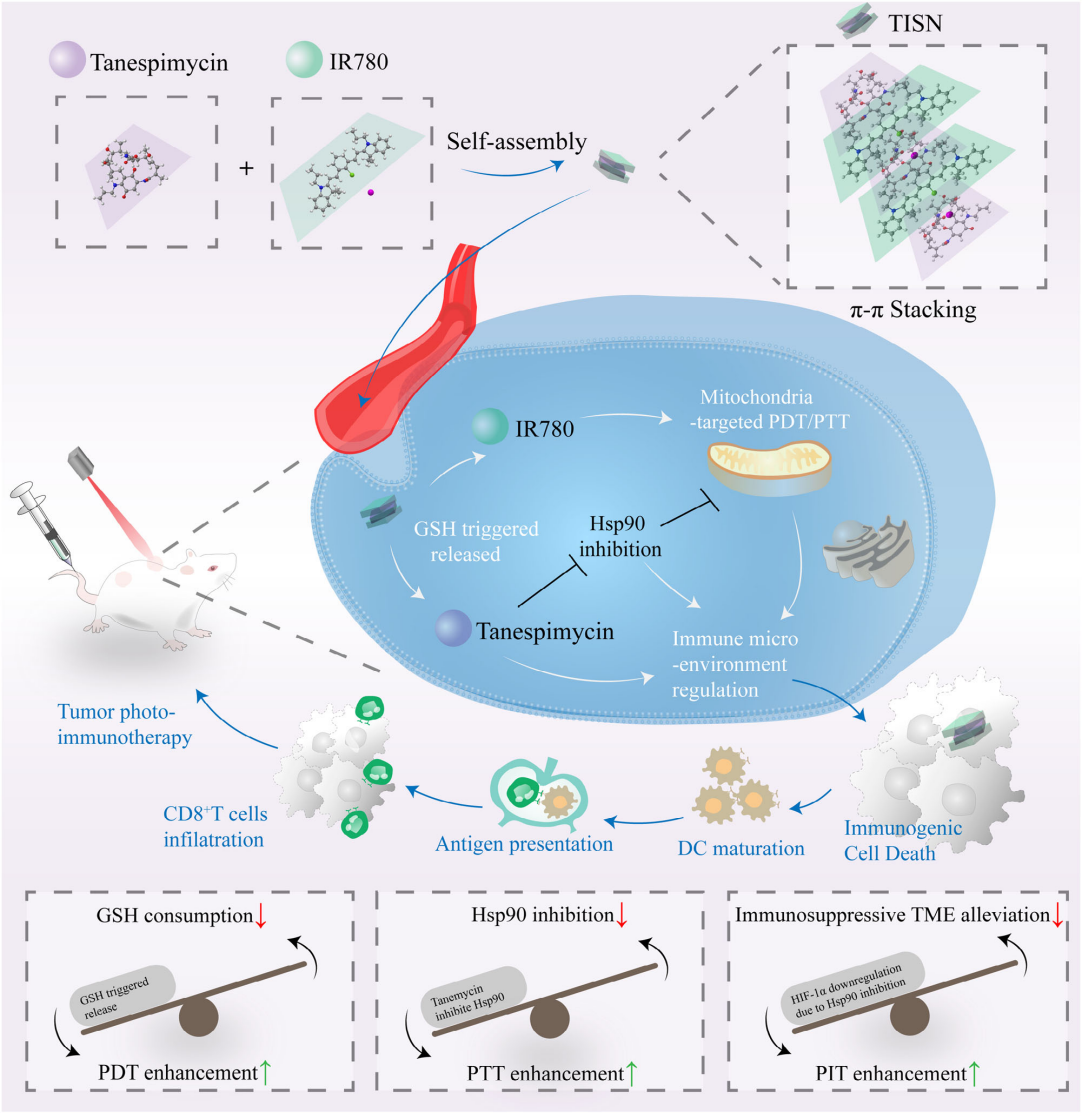
Schematic illustration of the design, synthesis, and functions of photo immune simulator. Such synergistic Mt‐PIT strategy simultaneously inhibited HSP90 to enhance PTT and led to GSH consumption to amplified PDT, manipulating tumor immunosuppressive microenvironment, and ultimately inducing local and distant tumor inhibition by mitochondria‐targeted immunotherapy.

Here, we have crafted a full API‐driven strategy to enhance mitochondria‐targeted PIT (Figure 1). This multilevel anticancer therapeutic approach encompasses the depletion of GSH, inhibition of Hsp90, immunosuppressive microenvironment alleviation, and Mt‐PIT. Notably, we found that Tanespimycin and IR780 are capable of self‐assembling into nanoparticles at a specific ratio through π–π stacking interactions, for both of them display macrocyclic structures. IR780, a cationic and lipid‐soluble dye with mitochondria‐targeting properties, serves as a versatile agent for both PDT and PTT. We have developed a novel Tanespimycin‐IR780 self‐assembly nanosystem (TISN), characterized by a 100% active pharmaceutical ingredient (API) content, designed for single‐dose, organelle‐specific therapy under the guidance of a single laser irradiation. This system is poised to advance the frontier of cancer treatment by combining the precision of targeted therapy with the power of multimodal phototherapy. We are confident that the TISN amplifies the dual‐mode phototherapy by leveraging intracellular reducing substances, Hsp90 inhibition, and mitochondrial dysfunction, thereby ultimately triggering a systemic anticancer immune response. Furthermore, TISN has the potential to emerge as a novel immune modulator for solid tumors, addressing a critical need in overcoming therapy resistance across the spectrum of treatments, including but not limited to PDT, PTT, and immunotherapy.

## 2 RESULTS

### 2.1 Characterization of TISN

**FIGURE 2:**
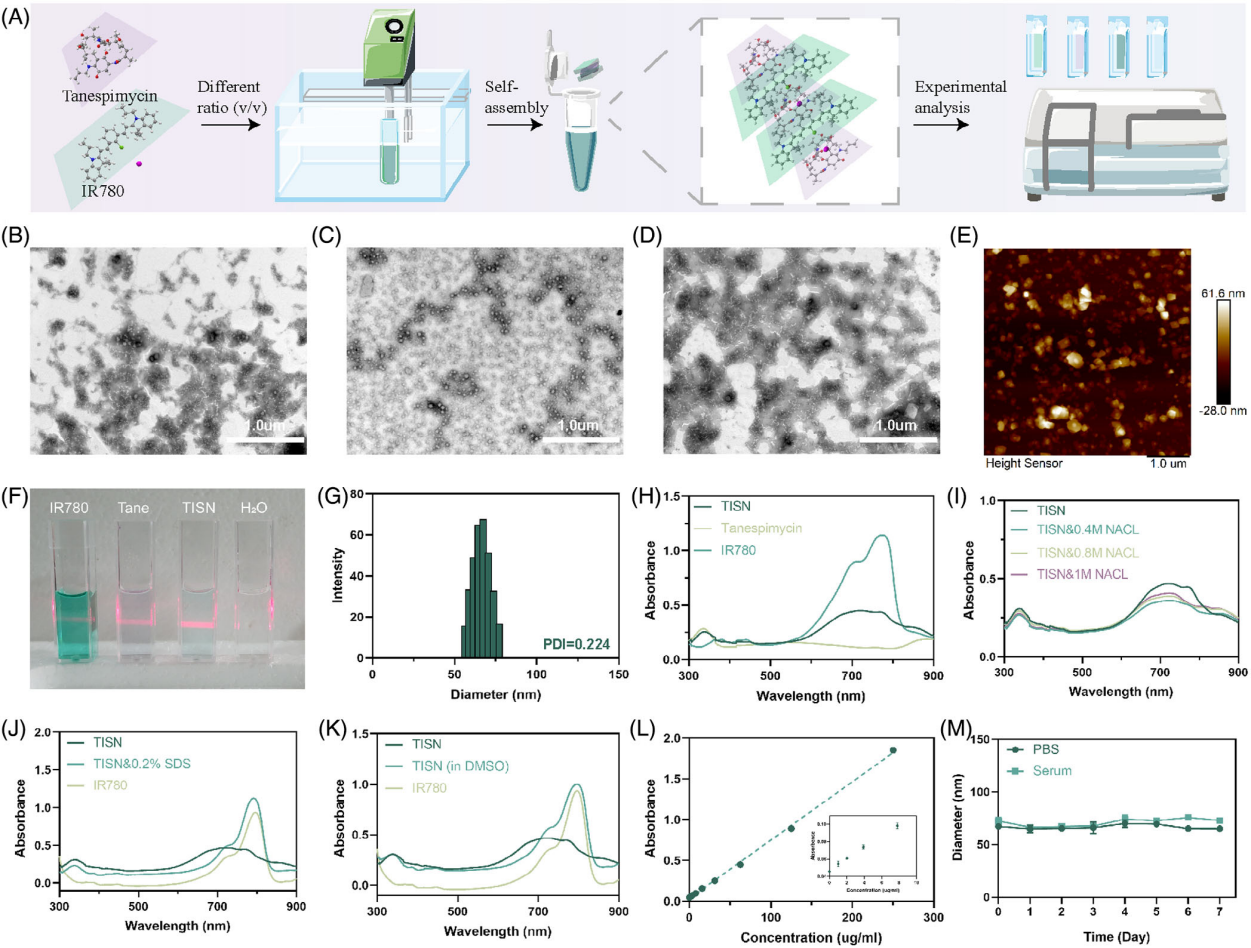
Preparation and characterization of TISN. (A) Schematic illustration of the preparation of TISN. (B–D) TEM images of different ratios of Tanespimycin and IR780, in which the proportion of 1:1 led to the construction of stable nanoparticles. The scale bars are 1000 nm. (B) The TEM image of Tanespimycin and IR780 with a 3:1 ratio. (C) The TEM image of Tanespimycin and IR780 with a 1:1 ratio. (D) The TEM image of Tanespimycin and IR780 with a 1:3 ratio. (E) The AFM image of TISN. (F) The image of the light path diagram of IR780, Tane, TISN, H_2_O. (G) The hydrodynamic diameters and PDI value of TISN. (H) UV–vis spectra of TISN, Tanespimycin, IR780. (I) UV–vis spectra of TISN with/without NaCl. (J) UV–vis spectra of IR780 and TISN with/without SDS (0.2%, w/v). (K) UV–vis spectra of IR780 and TISN in the presence and absence of DMSO. (L) Standard curves of TISN NPs. The inserted image displayed the data in low concentrations. (M) The stability of TISN in PBS and serum within 7 days.

TISN was synthesized according to Figure 1. Briefly, a heat shock protein inhibitor (Tanespimycin, Tane for short) and a photosensitizer (IR780) could self‐assemble into uniform nanoparticles (Figure 2A). Since both Tanespimycin and IR780 have large conjugated electron clouds, they are expected to form nanoparticles through π–π stacking interaction, and the self‐assembled nanoparticles were supposed to be obtained by adjusting the feed ratios of Tanespimycin and IR780.^26^ To verify this hypothesis, their morphologies were observed by TEM. The particles with a 3:1 (Figure 2B) or 1:3 (Figure 2D) ratio displayed high morphological heterogeneity, while the 1:1 group (Figure 2C) exhibited a homogeneously dispersed spherical morphology, with a 67.30 nm hydrodynamic size detected and a 0.224 PDI value by dynamic light scatter (DLS), which was selected for further analysis (Figure 2G). To further explore this interesting phenomenon, we observed the detailed morphology of TISN by atomic force microscope (AFM) and found the uniform multilayer structures, maybe indicating a potential fabrication mechanism via π–π stacking^27^ (Figure 2E). In Figure 2F, Tyndall's effect occurred in the TISN solution and further verified the nanostructure of TISN.

Moreover, we delved into the self‐assembly mechanism of TISN, employing a diverse array of investigative techniques to elucidate the underlying processes. Figure 2H demonstrated the absorption spectra TISN, Tane, and IR780. TISN exhibited a wider peak due to the π–π stacking and hydrophobic interaction between the aromatic ring of Tanespimycin and IR780.^28^ In Figure 2I, with the increase of sodium chloride ion strength in the solution, the UV–vis spectrum of TISN did not change significantly, indicating that the electrostatic interaction between molecules can be ignored. To delve deeper into the self‐assembly mechanism, TISN was dispersed in solutions of 0.2% (w/v) sodium dodecyl sulfate (SDS; Figure 2J) and DMSO (Figure 2K) for subsequent UV–vis spectroscopy analysis. After dissolving in DMSO, the typical absorption peak of IR780 in TISN was restored due to the destruction of noncovalent interaction between molecules. Moreover, a significant change was observed in the TISN spectrum in 0.2% SDS, as a result, SDS was involved in the hydrophobic effect in TISN. The absorption peak of IR780 in TISN is restored after the addition of SDS, which also confirms the important role of noncovalent bond interaction in TISN. Moreover, we recorded the FTIR spectrum of IR780 (Figure S1), Tanespimycin (Figure S2), and TISN (Figure S3). TISN displayed a strong peak at 2923.57 cm^−1^ characteristic of the intermolecular hydrogen bond. Additionally, a peak was observed at 1718.92 cm^−1^ characteristic of the conjugated bond. Moreover, the characteristic peaks at 1487.68 and 1025.62 cm^−1^ are indicative of C‐C skeleton stretching vibration and C‐O stretching vibration. These observations, coupled with the hydrophobic interactions and π–π stacking effects identified, suggest that hydrophobicity and π‐π stacking are the primary driving forces behind the self‐assembly of TISN.^28^ Different concentrations of TISN were used to draw the standard curve according to the absorbance at 780 nm (Figure 2L). The fluorescence spectrum of TISN was recorded in the Supporting Information (Figure S4). Additionally, the diameters of TISN aggregates in PBS and serum were monitored daily using DLS, and it was observed that they exhibited stability over a period of 7 days (Figure 2M).

### 2.2 GSH‐stimulated released and tumor penetration of TISN

**FIGURE 3:**
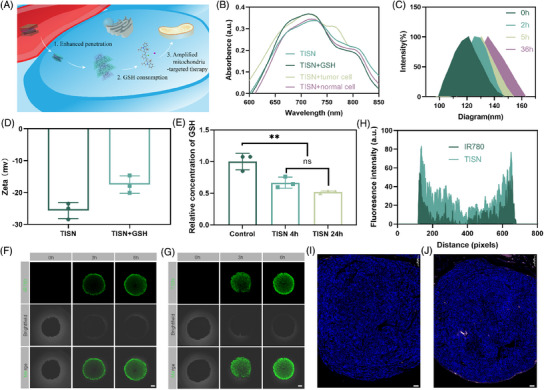
GSH consumption and tumor penetration of TISN. (A) The GSH consumption and tumor penetration behavior of TISN. (B) UV–vis spectra of TISN, TISN+GSH, and TISN coincubated with tumor cells. (C) DLS analysis of TISN incubation with GSH for 0, 2, 5, and 36 h. (D) ζ potential of TISN and TISN+GSH (*n* = 3). (E) Relative concentration of GSH in different GSH solutions, including the control group, TISN 4 h, and TISN 24 h (*n* = 3). (F) CLSM images of 3D tumor cell spheroids coincubated with IR780 for 0, 3, and 6 h. The scale bar is 100 µm. (G) CLSM images of 3D tumor cell spheroids coincubated with TISN for 0, 3, and 6 h. The scale bar is 100 µm. (H) Fluorescence analysis of Figure 2F,G by ImageJ. (I). Representative images of tumors from AGS‐bearing mice 24 h after injection of IR780. The scale bar is 100 µm. (J). Representative images of tumors from AGS‐bearing mice 24 h after injection of TISN. The scale bar is 100 µm.

Due to the competition of intracellular reducing agent GSH, the π–π stacking may be destroyed and thus lead to loading drug release in tumor cytoplasm.^29^, ^30^ Once TISN penetrated tumor cells, part of the TISN consumed the intracellular GSH and thus increased the ROS generation via mitochondria‐targeted PDT^31^ (Figure 3A). To verify this hypothesis, we performed a variety of in vitro and in vivo experiments. In Figure 3B, IR780 fluorescence was partly quenched after self‐assembly into TISN in the absence of GSH. After GSH was added, the fluorescence was recovered. It is well established that tumor cells exhibit a higher concentration of GSH compared with their normal counterparts. After incubation with AGS cells, the IR780 fluorescence appeared indicating the stimulated released behavior of TISN. Furthermore, TISN was observed to depolymerize upon the addition of GSH, which is associated with alterations in its size (Figure 3C) and zeta potential (Figure 3D), highlighting the role of GSH in the self‐assembly process of TISN. Moreover, the GSH consumption also indicates the disassembly of TISN and the release of Tanespimycin. We also observed the recovery of IR780 fluorescence to demonstrate the release of Tanespimycin after cellular uptake of TISN (Figures S5 and S6). After coincubation with IR780 and TISN for 0.5 h, the tumor cells were washed three times and observed in 0.5, 1, 2, and 4 h. As shown in the IR780 group, the fluorescence was stable from 0.5 to 4 h. In the TISN group, as IR780 and Tanespimycin were released, the fluorescence of IR780 gradually enhanced. To further quantify the consumption of GSH, we evaluated the GSH concentration at different time points after TISN was added (Figure 3E). Using the GSH/GSSG quantification kit, we found that GSH concentration was significantly consumed once TISN was incubated for 4 h. Though GSH concentration gradually decreased within 24 h, the residual concentration (66.62%) showed no significant difference compared with that at 4 h (51.92%). The consumption curve of GSH by Tanespimycin, IR780, and TISN (Figure S7) and the GSH concentration in tumor cells after different treatments (Figure S8) also proved the GSH depletion by TISN. To detect the Intracellular GSH depletion by TISN, we used FreSHtracer to evaluate the change in GSH concentration in tumor cells.^32^ As depicted in Figure S9, there were few red fluorescence signals (F580) before 0 h. After coincubation with TISN for 4 h, the fluorescence signals of F580 increased, indicating the intracellular depletion of GSH by TISN.

Next, we assessed the tumor penetration ability of TISN in 3D AGS tumor cell spheroids.^33^ As described in Figure 3F, after 3 and 6 h of incubation, IR780 was mainly located in the margin areas of the tumor spheroids and indicated a limited ability to penetrate deeper into the tumor mass. However, upon incubation for 3 and 6 h, the green fluorescence of TISN was gradually distributed throughout whole spheroids. Besides, the surface plots also displayed a stronger signal of TISN in AGS tumor spheroids than IR780 (Figure 3H). Moreover, we detected the tumor penetration of IR780 (Figure 3I) and TISN (Figure 3J) in vivo. 24 h after tail injection of IR780 and TISN (200 µL, 100 µg/mL IR780, tail vein injection), we discovered an enhanced fluorescence signal from TISN concentrated at the tumor's core, underscoring the superior penetrating capability of this self‐assembled system. This deeper penetration is a significant advantage, as it suggests that TISN can effectively reach the inner regions of tumors. In conclusion, these studies confirm the proficient tumor‐infiltrating ability of TISN. Such characteristics are instrumental in enhancing drug delivery efficiency, which is paramount for advancing cancer treatment strategies.

### 2.3 PDT and PTT effect of TISN

**FIGURE 4:**
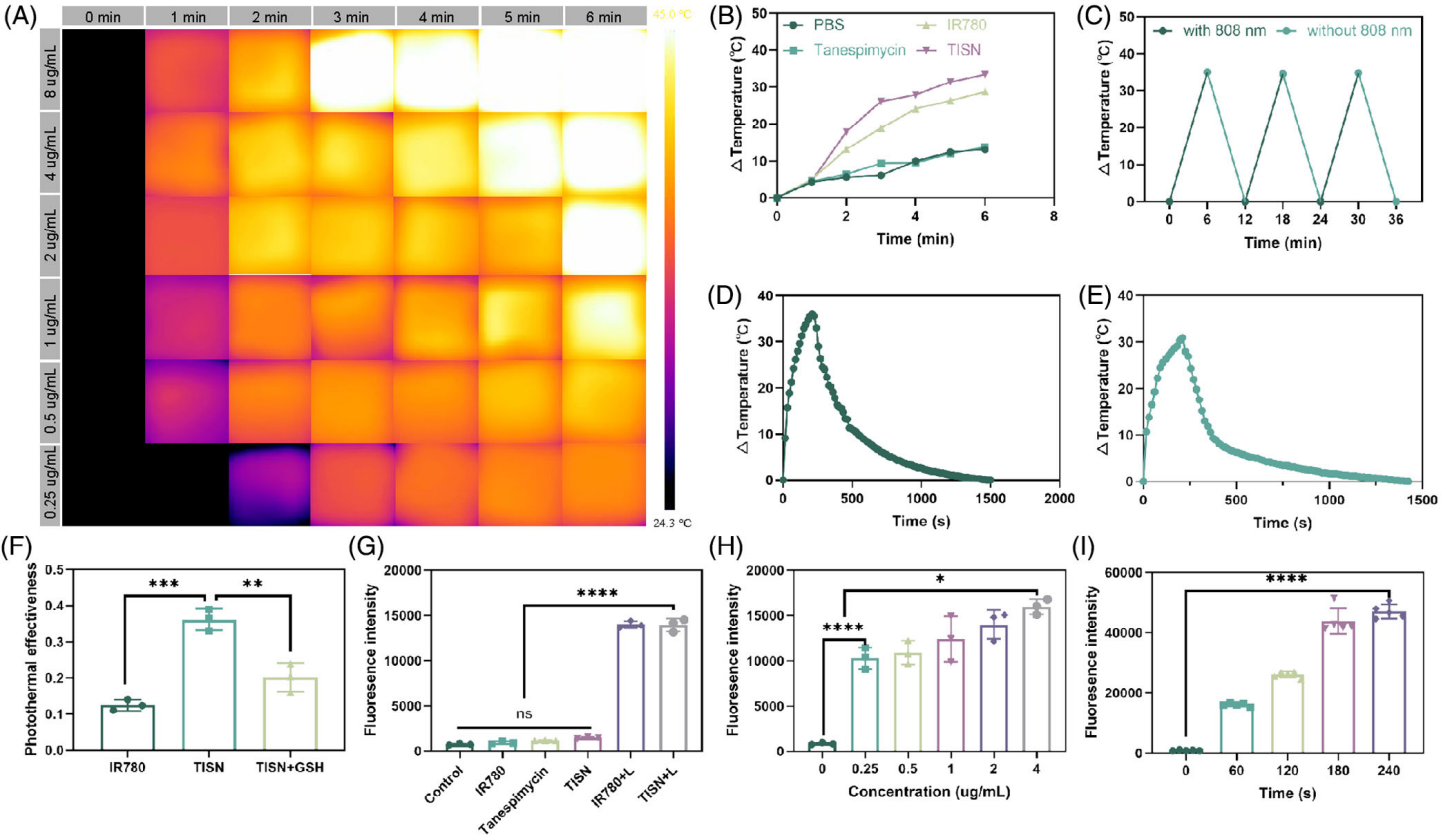
The photothermal and photodynamic ability of TISN. (A) Photothermal image of TISN at different dosage and irradiation time points. (B) The photothermal curve of PBS, IR780, Tanespimycin, and TISN. (C) Repetitive temperature change of TISN. (D) Temperature curve of TISN with 808 nm laser irradiation on for 210 s, followed by natural cooling. (E) Temperature curve of TISN with 808 nm laser irradiation on for 210 s, followed by natural cooling. (F) Calculated photothermal conversion efficiency of IR780 and TISN (*n* = 3). (G) The fluorescence signal intensity of SOSG in different groups (*n* = 3). (H) The fluorescence signal intensity of SOSG from TISN under 808 nm laser irradiation at different concentrations (*n* = 3). (I) The fluorescence signal intensity of SOSG from TISN under 808 nm laser irradiation at different time points (*n* = 3).

To verify the PTT effect of TISN, different concentrations of TISN solutions were prepared with sterile PBS, including 0.125, 0.25, 0.5, 1, 2, and 4 µg/mL, respectively. They were irradiated by 808 nm laser and image acquisition was carried out every 1 min with a thermal image. As shown in Figure 4A, which refers to its capacity to convert light into heat, has been observed to escalate with an increase in concentration. At the concentration of 0.125 µg/mL, laser irradiation for 6 min can only reach about 40°C. If the concentration reaches 1 µg/mL above, the temperature can reach higher than 50°C after 6 min’ irradiation. It is worth noting that in a concentration of 4 µg/mL, 3 min’ irradiation reached a high temperature.

To further investigate whether TISN exhibited a better photothermal effect than IR780, a thermocouple thermometer was utilized to measure different samples (PBS, 4 µg/mL Tane, 4 µg/mL IR780, 4 µg/mL TISN, the concentration of TISN was calculated according to IR780). In Figure 4B, the temperature curve of TISN and IR780 increases rapidly with the duration of laser irradiation. The temperature increased by more than 25°C in 3 min and increased by about 35°C in 6 min. The temperature changes in PBS and Tanespimycin groups were much smaller. The cyclic heat generation effect of TISN was also detected (Figure 4C), in which TISN recovered to room temperature after irradiation for 3 min, and continued another irradiation for 3 min. After several irradiation‐cooling loops, TISN can still maintain the effect of photothermal for 3 min to raise the temperature to about 35°C. Moreover, the photothermal conversion efficiency (PCE) of TISN was shown. We drew the temperature curve of TISN (Figure 4D) and IR780 (Figure 4E) in water with an 808 nm laser for 210 s followed by natural cooling. According to our previous study,^34^ we calculated the PCE of TISN, IR780, and TISN+GSH (Figure 4F). Due to unfavorable dispersal in water, the PCE of IR780 was 12.47%, while the PCE of TISN significantly increased and reached 36.16%. Once GSH was introduced, the nanostructure of TISN was destroyed and the PCE decreased to 20.14%. These data indicated that TISN could serve as a favorable PTT agent.

The excellent PTT effect of TISN inspired the exploration of its potential in PDT. As suggested in Figure 4G, the singlet oxygen sensor green (SOSG) signal remained almost unchanged in the absence of photosensitizer of laser irradiation, indicating a negligible generation of singlet oxygen (^1^O_2_), like the blank control. Once the laser irradiation was introduced, TISN illustrated a similar fluorescence increase to IR780, displaying a dependence of ^1^O_2_ production on constant illumination. Moreover, the fluorescence signals increased with the concentration of TISN (Figure 4H) or irradiation time (Figure 4I), indicating that TISN generated ^1^O_2_ under NIR laser irradiation in a dose‐dependent manner.

### 2.4 Synergistic mitochondria‐targeted phototherapeutic efficiency of TISN

**FIGURE 5:**
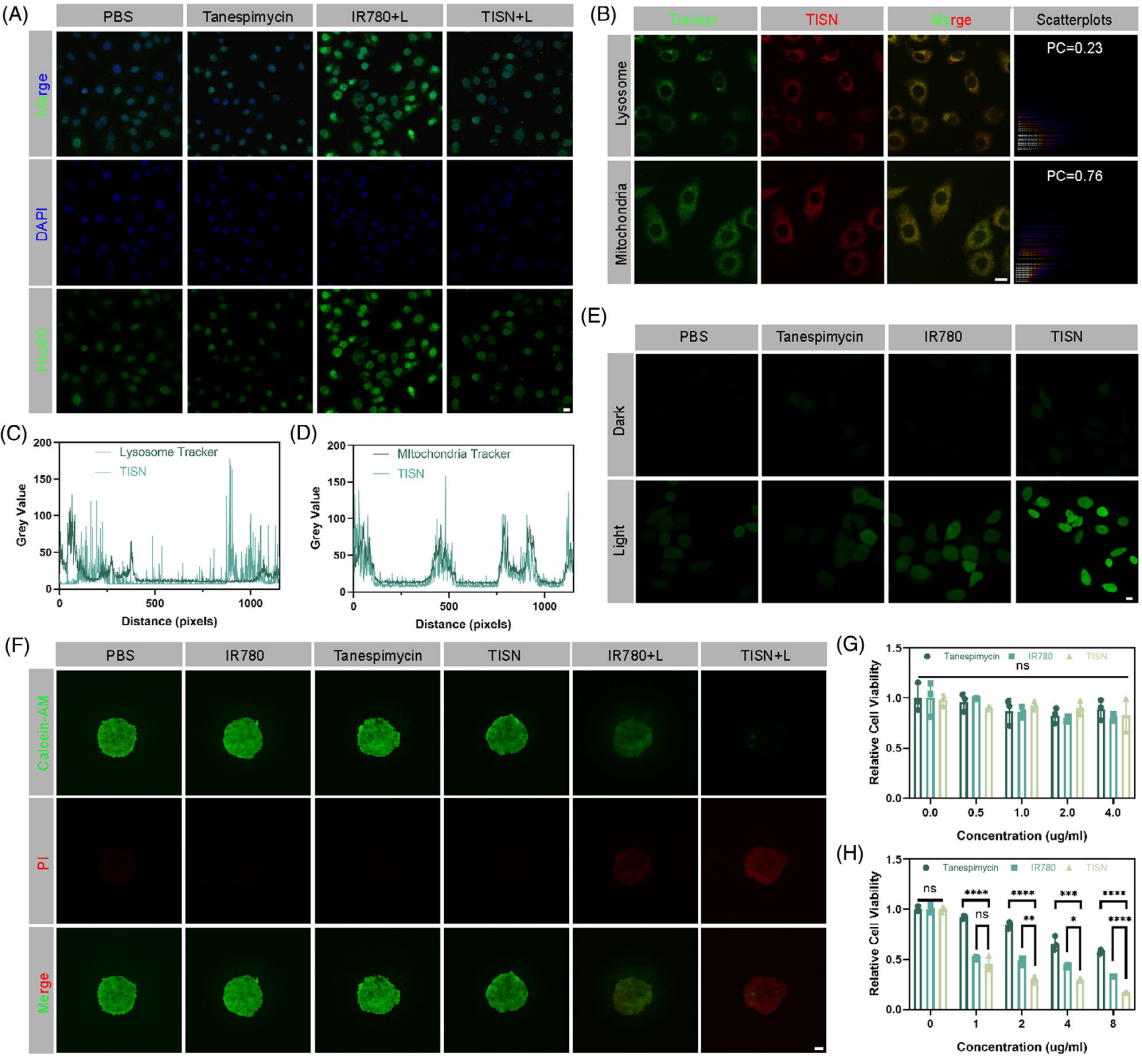
Amplified phototherapeutic effect by Hsp90‐inhibited and mitochondria‐targeted strategy. (A) CLSM images of Hsp90 staining (PBS, Tanespimycin, IR780+L, and TISN+L). The scale bar is 20 µm. (B) CLSM images of subcellular localization of TISN, lysosome, and mitochondria trackers. The scale bar is 10 µm. (C) Colocalization analysis of TISN and lysosome tracker. (D) Colocalization analysis of TISN and mitochondria tracker. (E) CLSM images of ROS production (PBS, Tanespimycin, IR780, and TISN with or without 808 nm laser irradiation). The scale bar is 20 µm. (F) CLSM images of cell spheroids (PBS, IR780, Tanespimycin, TISN, IR780+L, and TISN+L). The scale bar is 100 µm. (G) Relative cell viability without 808 nm laser irradiation (*n* = 3). (H) Relative cell viability with 808 nm laser irradiation (*n* = 3).

Tanespimycin inhibited Hsp90 and was applied to destroy the intracellular resistance against PTT and improve curative effect.^35^, ^36^ Moreover, HIF1α is a client protein of Hsp90 that could be downregulated by Tane.^25^, ^37^ Thus, the combination of Tanespimycin and mitochondria‐targeted photosensitizer IR780 was supposed to achieve strong photocytotoxicity against tumor cells. As described in Figure 5A, we detected the Hsp90 expression level in tumor cells under PTT by immunofluorescence. As a molecular chaperone, Hsp90 is present in various tumor cells for fast proliferation and hypermetabolism under normal conditions. Moderate green fluorescence was witnessed in the control group. Comparatively, in the presence of Tane, the weak green fluorescence of the Tanespimycin group indicated that the expression of Hsp90 was inhibited. However, a strong fluorescence signal was discovered in the IR780+L group, and the Hsp90 expression level was upregulated under stress conditions. As expected, the TISN+L group exhibited a low intensity of green fluorescence of Hsp90, suggesting the destruction of the intracellular defense against PTT.

We previously reported that IR780 could specifically combine with mitochondria.^14^, ^31^ We evaluated the subcellular localization and TISN. As illustrated in Figure 5B, different subcellular localization between the green fluorescence of lysosome and the red fluorescence of TISN. On the contrary, the red fluorescence exhibited almost the same subcellular localization as the green fluorescence of mitochondria. According to the colocation scatterplots, the Pearson Correction (PC) coefficient of TISN and lysosome was 0.23, but the PC coefficient of TISN and mitochondria was 0.76. Additionally, the colocalization analysis of lysosome tracker and TISN suggested a different trend (Figure 5C), but mitochondria tracker and TISN indicated a similar trend (Figure 5D). Furthermore, biological transmission electron microscopy was applied to analyze the subcellular localization of TISN in the revised manuscript (Figure S10). Treatment of tumor cells with TISN+L leads to conspicuous damage to mitochondrial structures. Moreover, we employed the JC‐1 assay kit to demonstrate the impact of various treatments on mitochondrial function within tumor cells, as depicted in Figure S11. Typically, under normal physiological conditions, mitochondria display red fluorescence, signifying a high mitochondrial membrane potential. In the control groups, which included treatments with PBS, IR780, Tanespimycin, and TISN, the majority of mitochondria preserved a healthy membrane potential, showing the persistent red signal. However, in the IR780 group, we discovered a significant decrease in red signals. Conversely, the TISN+L group displayed green fluorescence, a hallmark of a disrupted mitochondrial membrane potential. Based on the above results, we could conclude that the TISN showed specifically mitochondria‐targeted intracellular localization.

After proving the Hsp90 inhibition and mitochondria‐targeted features of TISN, the Mt‐PIT effect was further examined. After treatment with PBS, IR780, Tanespimycin, and TISN with or without 808 nm laser irradiation, H_2_DCFDA was utilized to detect ROS production (Figure 5E). In the PBS, IR780, and Tanespimycin without 808 nm laser irradiation, there was nearly no green fluorescence. In PBS+L, Tanespimycin+L, and IR780+L groups, in which ROS generated for GSH consumption or laser irradiation, there was a moderate level of ROS production. Compared with the IR780+L group, the TISN+L group showed the strongest green signal, indicating superior ROS production by enhanced PDT. Next, the synergistic mitochondria‐targeted phototherapeutic efficacy was measured by Calcein‐AM/PI double staining (Figure 5F). In the 3D spheroid tumor cells after different treatments, most of the living cells (marked as green) were found in the PBS, IR780, Tanespimycin, and TISN group, suggesting little cytotoxicity. Compared with the IR780+L group, a higher proportion of dying cells (red) in the TISN+L group were observed, suggesting a better curative effect. CCK‐8 kit was also applied for TISN treatment examination in different concentrations. In the absence of 808 nm laser irradiation, the relative cell viability showed no significant difference, suggesting a favorable biocompatible out‐of‐laser‐directing tumor area (Figure 5G). In Figure 5H, the relative cell viability of TISN with an 808 nm laser was significantly lower than the other two groups. The IC50 of Tanespimycin is 2.1331 nM while the IC50 of TISN is 0.7361 nM. We could conclude that TISN demonstrated both superior biosafety and potent phototherapeutic effect in the synergistic way of Hsp90 inhibition and GSH consumption.

### 2.5 Biodistribution and enhanced photothermal efficiency of TISN in vivo

TISN was able to be tracked by real‐time NIR equipment for biodistribution evaluation due to the outstanding NIR imaging property of IR780 in vivo.^38^ The mice injected with IR780 via tail intravenous were set as the control. The fluorescence of TISN was first detected at the tumor site by 6 h after tail intravenous injection of TISN, and the peak value was observed at 24 h after injection (Figure S12A). The fluorescence aggregated continuously on the tumor site, suggesting a considerable accumulation of TISN for the enhanced permeating and retention (EPR) effect. The fluorescence signals of TISN were stronger than IR780 due to deeper tumor penetration (Figure S12B). Ex vivo fluorescence results were reflected in Figure S13. A preferential accumulation of TISN was observed in tumor tissues rather than other organs. These different biodistribution profiles indicated that TISN exhibited an optimized tumor accumulation and prolonged circulation time, which may account for the improved tumor penetration after self‐assembly.

We used an infrared thermal imager to analyze the photothermal effects of TISN in vivo (Figure S12C). Tumor‐bearing mice in IR780+L and TISN+L groups were irradiated by 808 nm laser 24 h after intravenous injection. Different groups (Figure S12D) showed that the temperature of tumors in the IR780+L and TISN+L groups exhibited a mild temperature PTT effect. The temperature of tumors treated with other groups nearly did not change.

### 2.6 In vivo Hsp90 inhibition and antitumor efficacy

**FIGURE 6:**
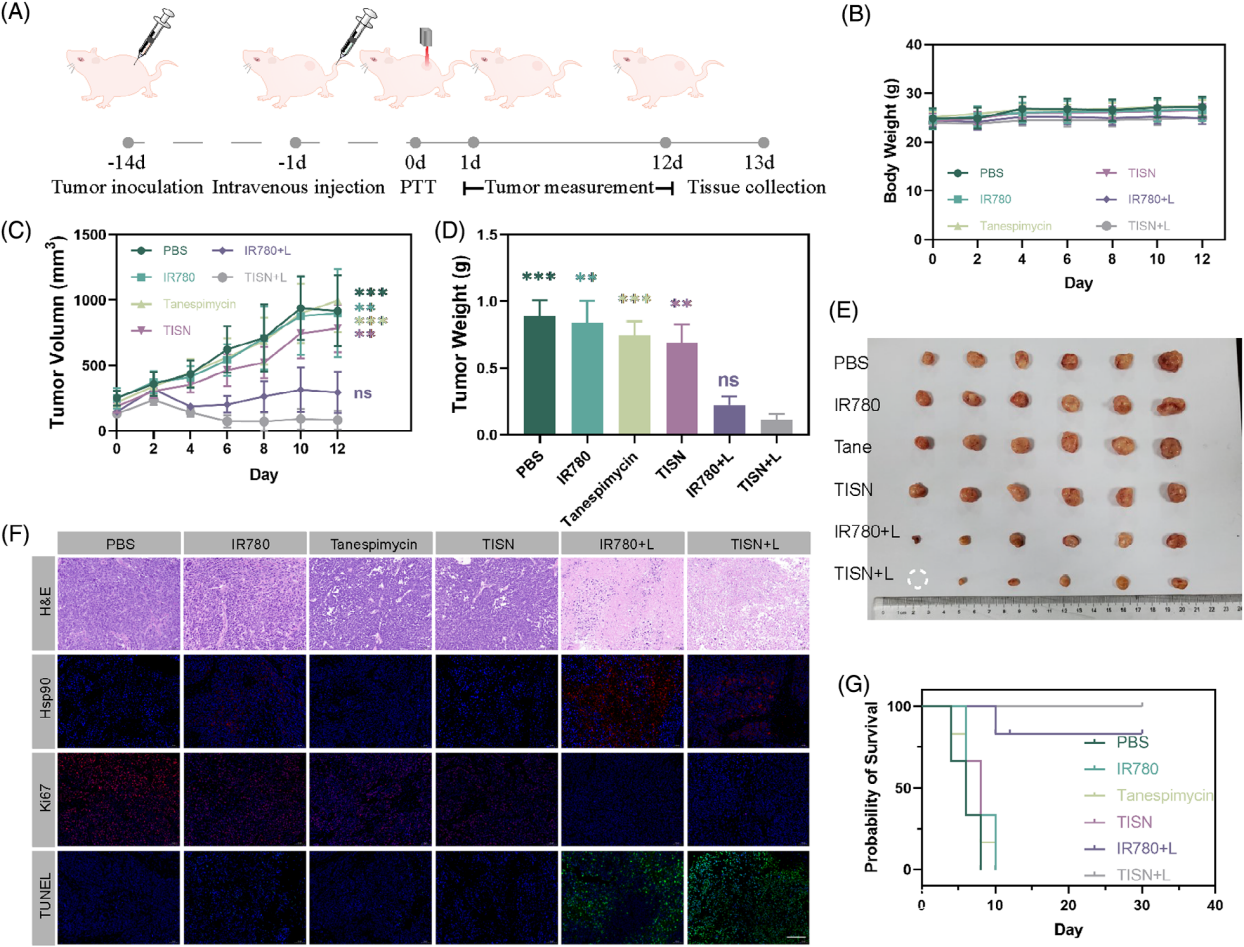
In vivo therapeutic effect of the TISN. (A) Schematic illustration of the treatment (1 W/cm^2^, 3 min) schedule after tail vein injection (200 µL, 100 µg/mL IR780, *n* = 6). (B) Body weight curve of different groups. (C) Tumor volume curves. (D) Tumor weights. (E) Photos of the tumor of AGS‐bearing mice after different treatments (PBS, IR780, Tanespimycin, TISN, IR780+L, TISN+L). (F) H&E, Hsp90, Ki67, and TUNEL staining tumor sections. The scale bar is 50 µm. (G) Survival curve.

Next, the dual‐mode phototherapeutic effect of TISN was examined. Tumor‐bearing mice in different groups (PBS, IR780, Tanespimycin, TISN, IR780+L, and TISN+L) were randomly assigned and subsequently evaluated the efficacy of TISN (Figure 6A). No significant differences in body weight were observed among the groups (Figure 6B), suggesting no significant acute toxicity. Tumor volume curves were demonstrated in Figure 6C, upon irradiation, TISN‐mediated mitochondria‐targeted PTT/PDT dual mode phototherapy exhibited extraordinary treatment effect. Tumors in the TISN+L group gradually subsided and one of them was completely ablated without recurrence in the examination period. Ultimately, the mice were euthanized on day 13, and the tumors were subsequently excised, weighted (Figure 6D), and photographed (Figure 6E). Compared with the PBS group, TISN‐mediated PIT displayed a 95% inhibition rate toward primary tumors. Furthermore, histological assessments, including hematoxylin and eosin (H&E), Hsp90, Ki67, and terminal deoxynucleotidyl transferase dUTP nick‐end labeling (TUNEL) staining, were presented in Figure 6F. In the TISN+L group, the majority of cancer cells exhibited severe cellular damage, characterized by karyorrhexis, karyopyknosis, and karyolysis. The Hsp90 inhibition ability of TISN was also detected. Strong red fluorescence signals of Hsp90 were found in the IR780+L group, indicating a strong resistance in tumor cells against heat. However, the Hsp90 expression level in the TISN+L group was significantly inhibited, which led to a potent anticancer activity by way of Hsp90‐inhibited PTT. The long‐range antitumor effect was further verified in another survival analysis (Figure 6G). These above results demonstrated that the TISN exhibited a strong curative effect via Hsp90‐inhibited and mitochondria‐targeted synergistic phototherapy.

### 2.7 TISN mediated ICD and systemic antitumor immunity

**FIGURE 7:**
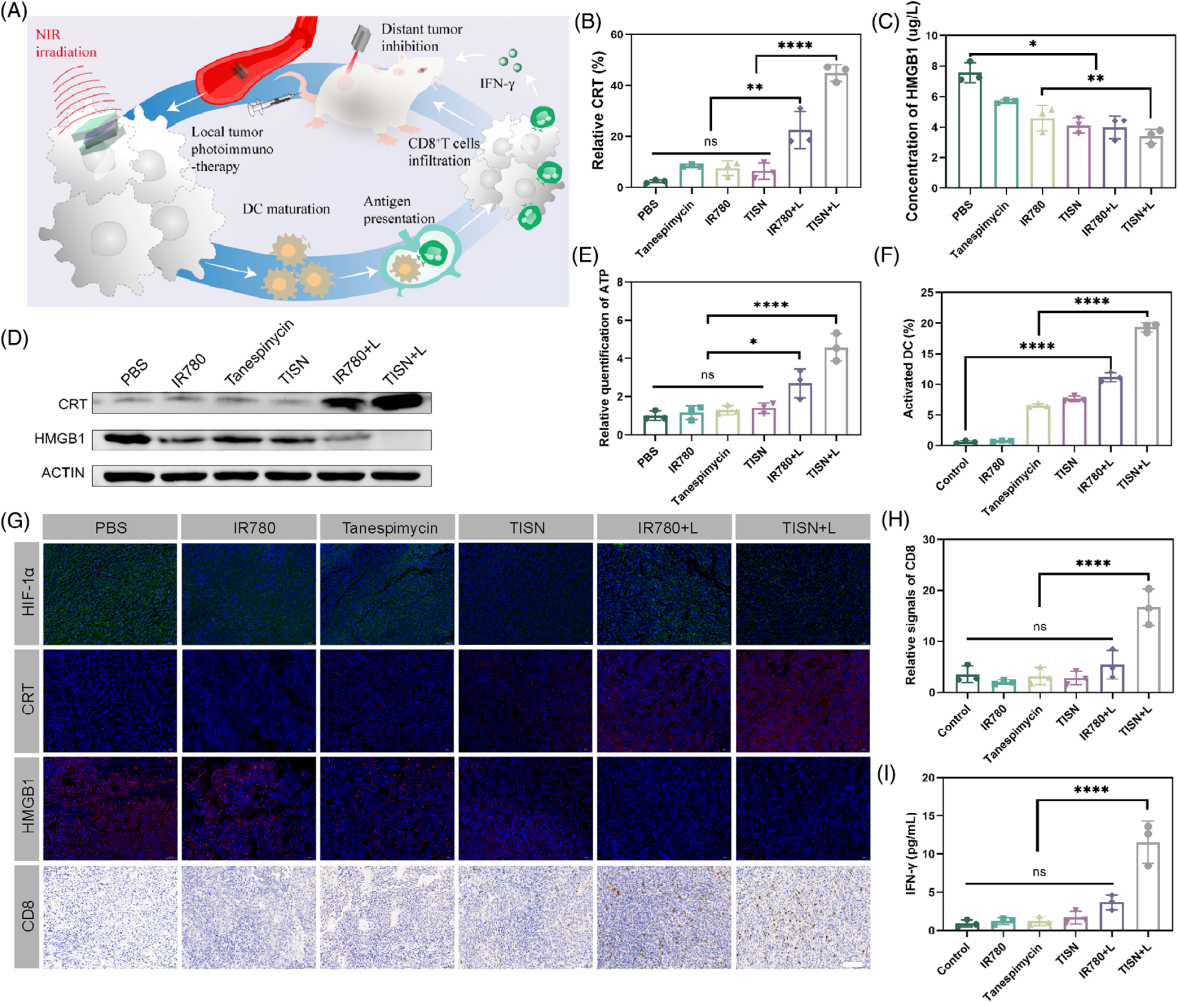
TISN triggered systemic anticancer immune responses through the ICD pathway. (A) Scheme illustration of ICD triggered by TISN. (B) Relative CRT level of CT26 cells (*n* = 3). (C) Concentration of HMGB1 in the supernatant of CT26 cells (*n* = 3). (D) Western blot analysis of the expression of CRT and HMGB1. (E) Relative ATP level (*n* = 3). (F) The proportion of matured DC in CT26‐bearing mice (*n* = 3). (G) The HIF‐1α, CRT, HMGB1, and CD8 staining in tumor slides. (H) The quantitative analysis of CD8 in Figure 7G (*n* = 3). (I) IFN‐γ level in the supernatant of CT26 cells (*n* = 3). The scale bar is 100 µm.

Since TISN‐based phototherapy exhibited the potential to prompt the activation of immune responses, we attempted to investigate ICD in TISN treatment^39^ (Figure 7A). With the introduction of TISN, the HIF‐1α level was significantly downregulated in TISN and TISN+L groups, as a result of Hsp90 inhibition (Figure 7B). CT26‐bearing mice treated with PBS, IR780, Tanespimycin, TISN, and IR780+L showed less exposure to calreticulin (CRT), but tumor cells treated with TISN in combination with laser irradiation exhibited significantly enhanced CRT expression on the cell surface, a key indicator of ICD. Quantitative analysis further substantiated the superiority of TISN over other treatments in inducing ICD, as evidenced by the pronounced cellular responses observed in the TISN group (Figure 7B,G). Compared with the groups treated with PBS, IR780, Tanespimycin, TISN, and IR780+L group, TISN+L group demonstrated a significantly enhanced release of high mobility group protein B1 (HMGB1) and ATP from CT26 tumor cells. This suggests that the TISN+L treatment notably potentiated the emission of key mediators associated with ICD (Figure 7C,E). Moreover, WB analysis of HMGB1 reflected the amplified HMGB1 release in CT26‐bearing mice of TISN+L treatment (Figure 7D). To elucidate the impact of DAMP release on dendritic cell (DC) maturation, we collected tumor‐draining lymph nodes from CT26 tumor‐bearing mice and performed flow cytometry analysis. As displayed in Figure 7F, treatment with IR780+L in colorectal tumor‐bearing mice elevated the proportion of mature DCs, characterized by CD80^+^ and CD86^+^ expression in CD11c^+^ DCs. Moreover, the TISN+L treatment markedly increased the ratio of mature DCs (CD80^+^ CD86^+^ CD11^+^) surpassing the effects observed in the IR780+L group. We previously found that highly immunogenic photosensitizers induced systemic antitumor immunity via CD8^+^T cell activation,^40^, ^41^, ^42^ thus we profiled the infiltrating CD8^+^T cells (Figure 7G). In the tumor microenvironment, the infiltration of CD8+ T cells was minimal in groups treated with PBS, IR780, Tanespimycin, TISN, and IR780+L, indicating that conventional phototherapy may be inadequate to modulate the immunological microenvironment effectively.^43^, ^44^ However, phototherapy sensitized with TISN+L notably intensified the infiltration of CD8+ T cells within the tumors (Figure 7H). Given that cytotoxic T lymphocytes can emit interferon‐γ (IFN‐γ), which is instrumental in modulating the tumor microenvironment, we proceeded to examine IFN‐γ in CT26 tumors in vivo (Figure 7I). The release of IFN‐γ in the TISN+L group was significantly more than in other groups, demonstrating its potential for optimal tumor immunotherapy.

### 2.8 Local and abscopal effects of TISN‐mediated synergistic PIT in vivo

**FIGURE 8:**
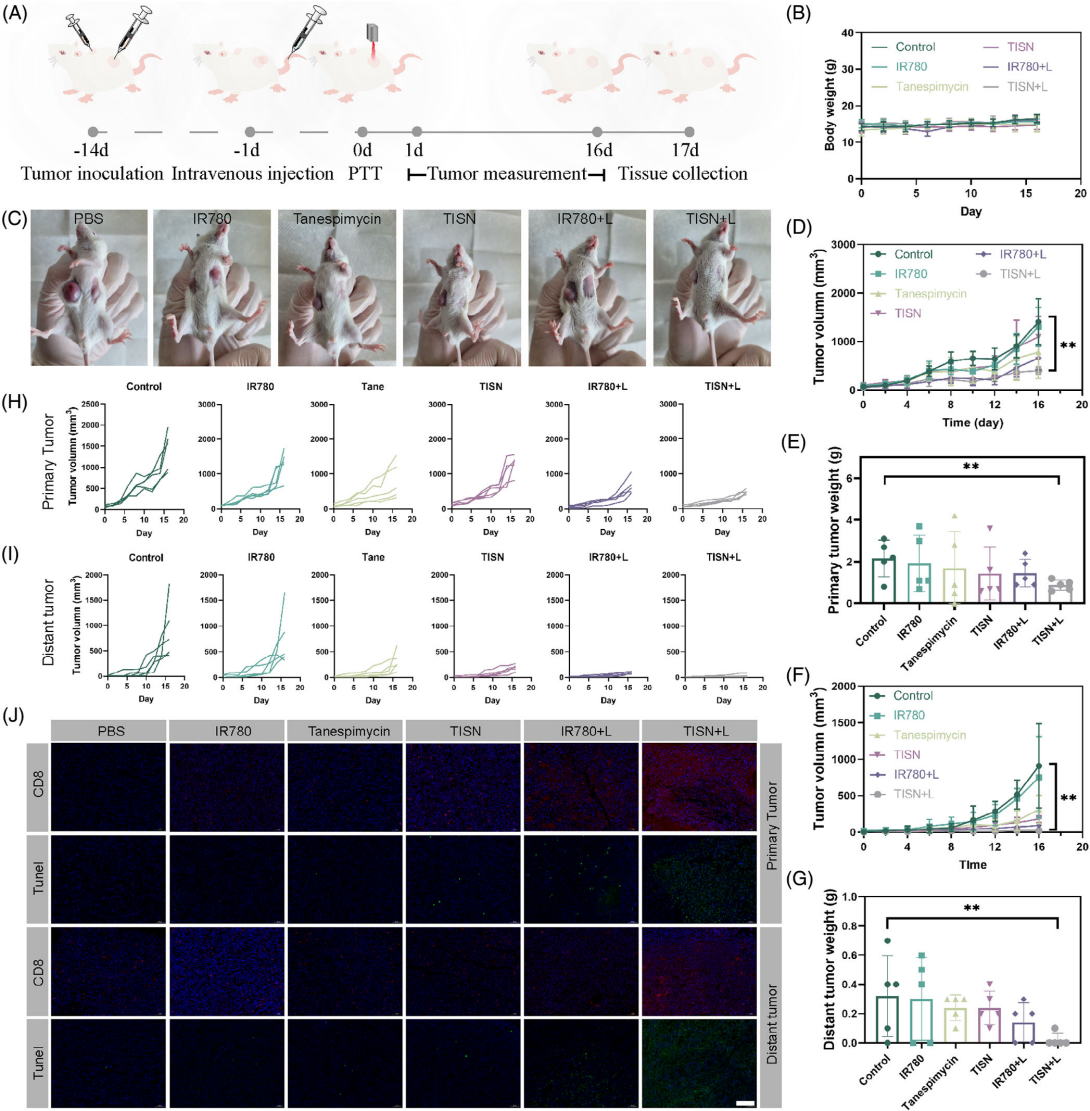
Antitumor immune responses against primary and distant tumors of the TISN. (A) Schematic illustration of the treatment (1 W/cm^2^, 3 min) schedule (200 µL, 100 µg/mL IR780, *n* = 5). (B) Body weight curve. (C) Represented images of the mice after different treatments (PBS, IR780, Tanespimycin, TISN, IR780+L, TISN+L). (D) Primary tumor volume curves. (E) Primary tumor weights. (F) Distant tumor volume curves. (G) Distant tumor weights. (H) Primary tumor volume curves of every mouse. (I) Distant tumor volume curves of every mouse. (J) H&E, Hsp90, Ki67, and TUNEL staining tumor sections with a scale of 50 µm.

We sought to examine whether this synergist PIT strategy could inhibit tumor metastasis (Figure 8A). The body weight of tumor‐bearing mice indicated there was no acute biotoxicity in this TISN‐mediated Hsp90 inhibition and mitochondria‐targeted synergist PIT strategy (Figure 8B). Remarkably, TISN+L treatment significantly inhibited primary tumor growth (Figure 8C,D,H). The tumors were collected on day 17 and weighed (Figure 8E). Furthermore, TISN‐mediated PIT also significantly decreased the growth of untreated abscopal tumors, and tumors in two mice were eradicated (inhibition rate: 97.4%; Figure 8F,I). The tumor weights further confirmed the superior anticancer effect of this synergistic PIT strategy (Figure 8G). Additionally, the immunofluorescence images of tumors demonstrated increased tumor destruction and CD8^+^ T cells infiltrating into both local and abscopal tumors after TISN+L intervention (Figure 8J). These results displayed the TISN‐mediated synergist PIT strategy could initiate a system immune response and generate an abscopal effect.

### 2.9 Biosafety evaluation

H&E staining was conducted on key organs, encompassing the heart, liver, spleen, lung, and kidney, to assess the safety (Figure S14A). In comparison with the control group, no significant signs of inflammatory lesions, hydropic degeneration, or histopathological necrosis were observed post‐treatment, underscoring the excellent systemic biocompatibility of TISN. Furthermore, hematological and serological assessments were undertaken to scrutinize the potential long‐term biosafety of TISN (Figure S14B–L). These findings underscore the high therapeutic biosafety of TISN and suggest its suitability for future clinical applications.

## 3 DISCUSSION

Phototherapy is widely applied in tumor treatment nowadays, while systemic anticancer immune response is rarely induced clinically.^45^ The lack of precise and potent photo‐immune stimulators remains a main challenge. Resistant mechanisms including intracellular reducing substances,^46^ heat shock proteins, and insufficient oxygen concentration retarded the PIT induced in tumors. Notably, human tumors demonstrate higher levels of GSH and Hsp90 than normal tissues, displaying the vital role of defense mechanisms in tumors against PIT. Mitochondria, the important subcellular organelles, serve as the driving force for the above resistant mechanisms by consuming oxygen to reduce ATP, in which the ROS generated would be decreased for insufficient oxygen and the Hsp90 was activated in an ATP‐dependent manner. Thus, the mt‐PIT we report here was achieved by a full‐API photo‐immune stimulator, with efficient mitochondria‐targeted and amplified dual‐mode PDT/PTT ability, ultimately leading to a systemic antitumor immune response through the ICD pathway. Moreover, mitochondria were proved to be essential for cancer proliferation and the establishment of an immunosuppressive microenvironment, but showed greater susceptibility to therapeutic inventions.^47^ Our study underscores the pivotal role of mitochondria and highlights their potential as an exemplary target for subcellular organelle‐specific immunotherapy

Recently, several PIT‐based strategies were proposed by physical, chemical, and biological strategies, but none of them were translated into the clinic. The major challenges for the limited efficacy of PIT were insufficient immune response triggered by phototherapeutic stimulation and unfavorable tumor immunosuppressive microenvironment. Therefore, the combination of dual‐mode PDT/PTT and resistance alleviation strategies was urgently needed. We previously reported various immune microenvironment‐regulated strategies, including enhanced T cell infiltration through platelet inhibition,^48^ bacteria‐mediated DC activation,^49^ and photosynthetic organism‐based PIT strategies,^50^ without regard to the inherent resistant mechanism of tumors. In this study, we focused on the precise downregulation of natural resistances to PIT, including redox homeostasis, heat shock proteins, and HIF‐1α, ultimately achieving an amplified PIT and superior anticancer treatment. Moreover, this PIT strategy was accurately performed in the tumor site due to the deep tumor penetration, mitochondria‐targeted ability, and NIR laser irradiation, thus triggering a precise anticancer immune response. Additionally, full‐API design by self‐assembly improved the API to 100% without ineffective payloads and led to better efficiency.

Subcellular, or organelle‐targeted therapy, enhances spatiotemporal selectivity and confers a heightened therapeutic efficacy.^51^, ^52^ Mitochondria are novel targets for organelle‐targeted therapy, for their critical roles in cell survival and proliferation.^53^ However, mitochondria, being sensitive to external stimuli, can induce cell dysfunction, apoptosis, and ICD upon damage.^54^ Because of the short half‐life of ROS, the action ranges of phototherapeutic inventions are usually limited and much smaller than the cell size (<20 nm), efficient PIT must hit the bull's eye. Mt‐PIT we reported here provided a novel and impactful strategy for tumor immunotherapy, making the best use of the advantages and bypassing the disadvantages of traditional PDT and PIT. Furthermore, the pharmacokinetics of nanoparticles are critical for targeting delivery.^55^ TISN exhibited optimized pharmacokinetics including enhanced tumor accumulation, deeper penetration, and less distribution in major organs, indicating the potential for clinical translation.

Moreover, all the components of TISN have demonstrated biocompatibility. Tanespimycin was an ICD inducer and had entered more than ten clinical trials. In phase I clinical trials, Tanespimycin exhibited low toxicity (e.g., NCT00019708, NCT00779428). However, several phase II clinical trials failed when Tanespimycin combated relapsed and refractory tumors as the monotherapy (e.g., NCT00104897, NCT00118428). Combination with a frequently‐used photosensitizer, TISN was self‐assembly without either a drug carrier or stabilizer, thus avoiding unexpected immune responses and potential toxicity. Safety concerns usually retard the clinical translation of novel immune agonists, while clinical trials validated drugs formed self‐assembly nanosystems may stride across the gap between basic study and application.^56^ There were several limitations such as lacking clinical trials, unconfirmed long‐term transportation stability, and needing more experiments to confirm its effectiveness. Nevertheless, our work, characterized by high safety and efficacy in tumor treatment, holds promise for future clinical applications.

## 4 CONCLUSION

In summary, full‐API TISN was fabricated from a phase II Hsp90 inhibitor and a mitochondria‐targeted photosensitizer for enhanced PIT featured with GSH consumption, Hsp90 inhibition, and mitochondria‐targeted ability. This simple synthesis system exhibited favorable stability, good biocompatibility, and deep tumor penetration. Moreover, the GSH‐triggered release characteristic of TISN enhanced PDT by GSH consumption and mitochondria‐targeted capability, while Hsp90 inhibition led to the destroyed resistance of tumor cells against PTT. This synergistic phototherapeutic intervention not only triggered cancer cell death but also evoked a robust ICD‐mediated systemic anticancer immune response. TISN promoted CRT translocation, HMGB1 release, and ATP secretion, thus activating matured DCs and improving CD8^+^ T cell infiltration. Armed with HIF‐1α downregulation by Tanespimycin, TISN reversed the immunosuppressive tumor microenvironment and ultimately achieved a superior inhibition in both local and abscopal tumors by this synergistic mitochondria‐targeted PIT strategy.^57^ Compared with our previously reported photo‐immune sensitizers,^11^, ^58^ this current technique focused on heat shock protein inhibition and enhanced synergistic tumor PIT by simply full‐API combination of clinical trial‐failed anticancer drugs and a novel photosensitizer. Such self‐assembly NPs as full‐API delivery systems could be an effective and safe photo‐immune stimulator. Moreover, this strategy also exhibited the potential to advance therapeutic interventions such as radiotherapy, chemotherapy, and targeted therapy, where the immunosuppressive status was still an important cause for clinical trial failure.

## 5 MATERIALS AND METHODS

### 5.1 Materials

Tanespimycin and H_2_DCFDA were purchased from MedChemExpress (Shanghai, China). IR780, Calcein AM, and PI were purchased from Sigma–Aldrich (Missouri, USA). DMSO was purchased from Sinopharm Chemical Reagent Co. DAPI and SOSG were purchased from Keygen Biotech. Anti‐CD11c, anti‐CD80, anti‐CD86, and anti‐CD8 antibodies were purchased from Proteintech.

### 5.2 Synthesis of TISN

TISN NPs were synthesized as previously reported. Briefly, Tanespimycin and IR780 were dissolved in 100 µL DMSO, respectively. Next, the two solutions at different volume ratios were mixed. Then pure water was added gradually under high‐speed dispersion to form TISN NPs. The resulting solution was put in a dialysis bag (1000 kDa) for 2 h, to remove free Tanespimycin and IR780.

### 5.3 Characterization

The morphology and size of TISN with different ratios of Tanespimycin and IR780 were observed by TEM (JEOL, Japan). DLS (90Plus; Brookhaven Instrum. Corp) was used to detect the particle diagram and zeta potential. The absorption spectrum was evaluated by UV‐2450 (Shimadzu, Japan).

### 5.4 Cells culture and animal model

AGS and CT26 cells were obtained from Pricella Biotechnology Co., Ltd at 37°C in 5% CO_2_. Cell construction and experiment were conducted once the cells reached 80% confluence. AGS cells were suspended in PBS (1 × 10^7^ cells in 100 µL each mouse) and then subcutaneously injected into the right upper extremity area of nude mice. Tumor volume was evaluated by (length × width × width × 0.5). CT26 colorectal bilateral cancer model was established by a similar method.

### 5.5 Measurement of penetration of TISN NPs

5 × 10^3^ per well of tumor cells seeded into ultralow attachment round‐bottom 96‐well plates to achieve 3D cell spheroids. While the spheroids formed, IR780 and TISN NPs were added and incubated for 0, 3, and 6 h, respectively. The penetration depth of IR780 and TISN NPs was detected by a confocal microscope.

### 5.6 Detection of the photothermal effect of TISN

To ascertain the photothermal capabilities of PBS, IR780, Tanespimycin, and TISN, a range of concentrations were utilized to evaluate the PTT effect across different groups. The in vitro PTT effect was quantified using a thermocouple thermometer (TAIS 600), with concurrent documentation of thermal images via a Visual IR Thermometer (FLUKE VT02). For in vivo assessments, a similar methodology was employed, utilizing the same Visual IR Thermometer. AGS tumor‐bearing mice were injected with 200 µL of the respective solutions at a concentration of 4 µg/mL IR780, with or without exposure to an 808 nm laser at an intensity of 1 W/cm^2^. Thermal images and temperature recordings were captured accordingly. The PCE of TISN was determined using the formula as described by Wang et al. Following a 24‐h incubation period with the aforementioned solutions, the expression of Hsp90 in AGS cells was analyzed through western blotting.

### 5.7 Subcellular localization of TISN and in vitro antitumor efficacy

Subcellular localization was conducted in the previous method. For the assessment of cell death using the Calcein‐AM/propidium iodide double staining method, cells were coincubated with 100 µL of the staining solution in confocal wells (20 mm glass‐bottom dishes; NEST Biotechnology Co. Ltd.) for 30 min. After this incubation, the cells were washed three times to remove excess stains and then examined using a confocal microscope. Live cells were stained with Calcein‐AM, while dead cells were identified by propidium iodide fluorescence. The quantitative analysis of stained cells was performed using ImageJ software.

### 5.8 In vivo biodistribution

After being injected with TISN (200 µL, 100 µg/mL IR780), NIR fluorescence images were acquired by the CRI maestro system (*λ*_ex_/*λ*_em _= 740/810 nm) at different time points (3, 6, 12, 24, and 36 h). Another group of mice was euthanized for fluorescence imaging of tumors and organs.

### 5.9 In vivo phototherapy and systemic antitumor immune effect

Mice were divided into PBS, IR780, Tane, TISN, IR780+L, and TISNL groups (*n* = 5). After treatment for 12 days, the mice were sacrificed and tumor tissue was collected for further histopathology analysis. In vitro and in vivo experiments were conducted as previous publication.^59^ Once the immune cells in the spleen were obtained, then resuspended into the 24‐well plate with 24 h pre‐seeded CT26 cells with 2 × 10^5^ cells per well. After a 72‐h incubation, the concentrations of TNF‐α and IFN‐γ in the supernatant were measured.

### 5.10 Statistical analysis

Statistical analyses were done via GraphPad Prism software with a significance level for ns *p* > 0.05, *p < 0.05, **p < 0.01, and ***p < 0.001. Data were shown in the mean ± standard deviation (SD) method.

## AUTHOR CONTRIBUTIONS

*Conceptualization, methodology, investigation, visualization, writing—original draft, writing—review and editing, funding acquisition*: Xianghui Li. *Methodology, investigation, visualization*: Haoran Wang. *Investigation, visualization*: Zhiyan Li. *Investigation, visualization*: Song Liu. *Investigation, visualization*: Yuanyuan Chen. *Investigation, visualization*: Zhijian Yao. *Investigation, visualization*: Zhuren Ruan. *Investigation, supervision*: Wenjun Zheng. *Investigation, writing—review and editing*: Cunwei Cao. *Conceptualization, supervision, writing—review and editing, funding acquisition*: Wenxian Guan. All authors have read and approved the final manuscript.

## CONFLICTS OF INTEREST STATEMENT

The authors declare that they have no conflict of interest.

## ETHICS STATEMENT

All animal tests and experiment procedures used in this experiment were performed in accordance with protocols approved by the Institutional Animal Care and Use Committee of Nanjing University (NJU‐IACUC, IACUC2003160). We confirmed all methods were carried out in accordance with relevant guidelines and regulations.

## Supporting information



Supporting Information

## Data Availability

We declare that materials described in the manuscript, including all relevant raw data, will be freely available to any scientist wishing to use them for noncommercial purposes. The datasets used and analyzed during the current study are available from the corresponding author upon reasonable request without breaching participant confidentiality.
