# Supplementary material for: Full‐active pharmaceutical ingredient nanosensitizer for augmented photoimmunotherapy by synergistic mitochondria targeting and immunogenic death inducing
Source: MedComm (2020). 2024 Nov 9;5(11):e756. doi: 10.1002/mco2.756 (PMC11550090; doi:10.1002/mco2.756)
Supplement: Supplementary file 1 — Supporting Information [file MCO2-5-e756-s001.docx]

Supporting Materials for

Full-Active Pharmaceutical Ingredient Sensitizer for Augmented Photoimmunotherapy by Synergistic Mitochondria Targeting and Immunogenic Death-Inducing

Xianghui Li^1,2, #, *^, Haoran Wang^3^^, #^, Zhiyan Li^2, #^, Song Liu^2^, Yuanyuan Chen^1^, Zhuren Ruan^1^, Zhijian Yao^1^, Gao Wei^1^, Cunwei Cao^1^, Wenjun Zheng^1, *^,Wenxian Guan^2, *^

1 The First Affiliated Hospital of Guangxi Medical University School, Nanning, 530021, China

2 Affiliated Nanjing Drum Tower Hospital, Nanjing University Medical School, Nanjing, 210008, China

3 State Key Laboratory on Technologies for Chinese Medicine Pharmaceutical Process Control and Intelligent Manufacture，Nanjing University of Chinese Medicine, Nanjing 210023, China

^#^Xianghui Li, Haoran Wang, and Zhiyan Li contributed equally.

*Corresponding to

* **Xianghui Li** – *Department of Dermatology and Venereology, The First Affiliated Hospital of Guangxi Medical University, Nanning, 530021, China; Email:* *doctorlixianghui@smail.nju.edu.cn*

* **Wenjun Zheng** – *Department of Dermatology and Venereology, The First Affiliated Hospital of Guangxi Medical University, Nanning, 530021, China; Email: gxmuzwj@163.com*

* **Wenxian Guan** – *Department of Gastrointestinal Surgery, Affiliated Nanjing Drum Tower Hospital, Nanjing University Medical School, Nanjing, 210008, China; Email: medguanwx@163.com*


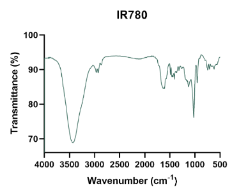


***Figure S1****. The FTIR spectrum of IR780.*


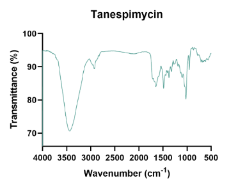


***Figure S2****. The FTIR spectrum of Tanespimycin.*


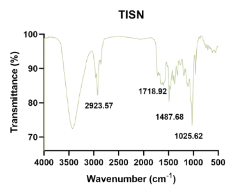


***Figure S3****. The FTIR spectrum of TISN.*


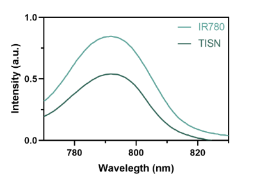


***Figure S4****. The fluorescence spectrum of TISN and IR780.*


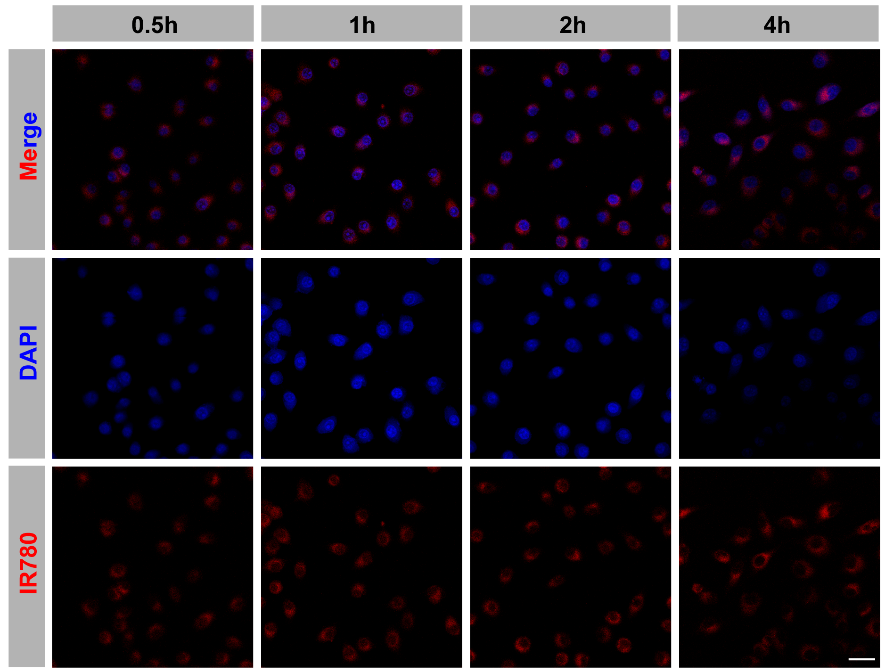


***Figure S5****. The CLSM images of tumor cells after co-incubation with IR780 in 0.5h, 1h, 2h, and 4h. The scale bar is 20μm.*


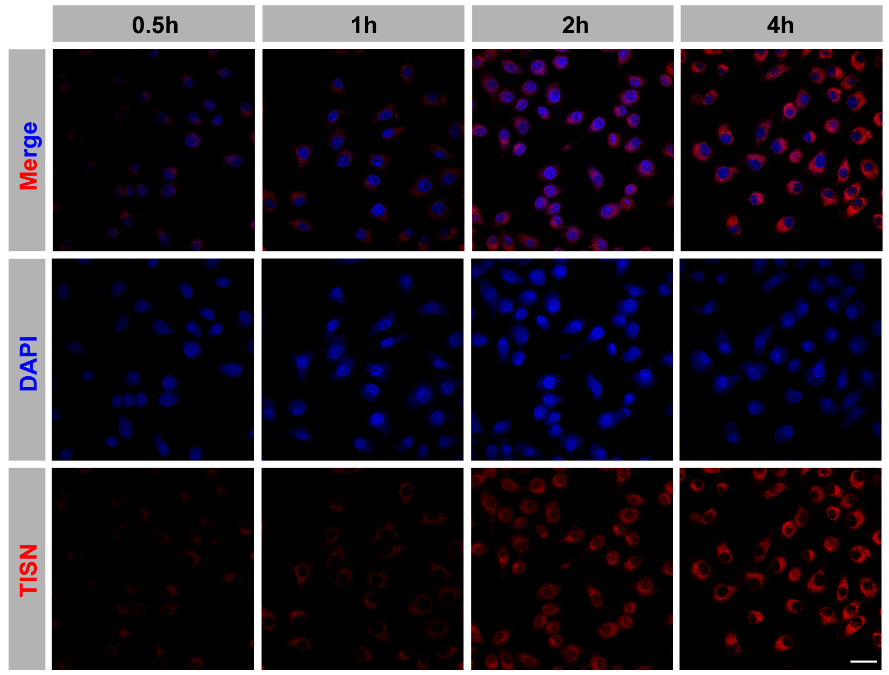


***Figure S6****. The CLSM images of tumor cells after co-incubation with TISN in 0.5h, 1h, 2h, and 4h. The scale bar is 20μm.*


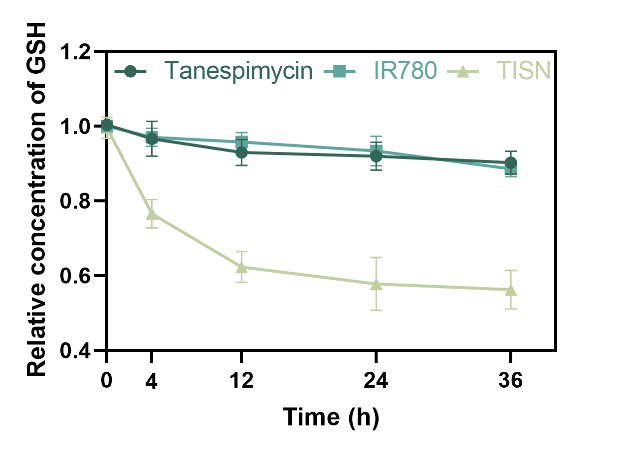


***Figure S7****. The GSH concentration curve in tumor cells co-incubation with Tanespimycin, IR780, and TISN in 0, 4, 12, 24, and 36h.*

***
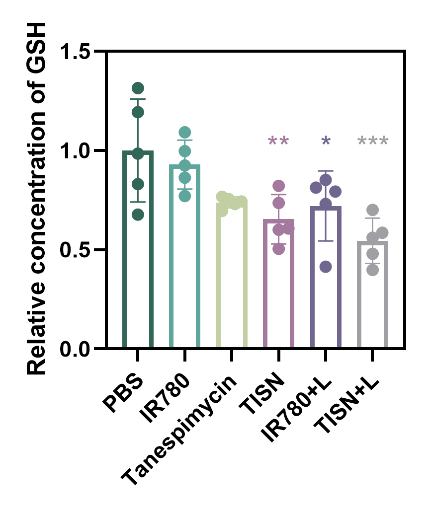
***

***Figure S8****. The* *GSH concentration in tumor cells after different treatments.*

*
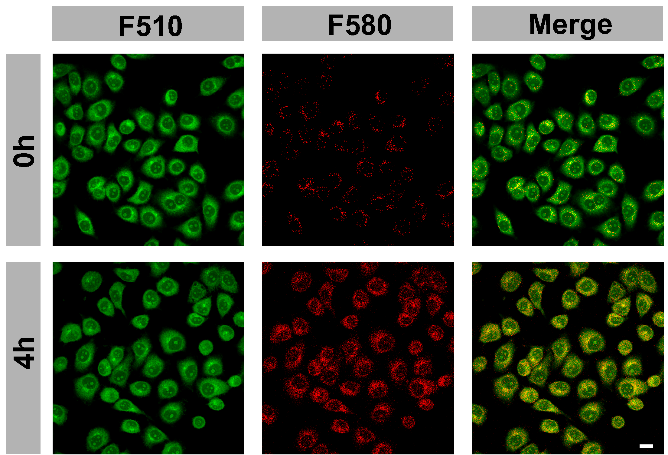
*

***Figure S9****. Intracellular GSH level evaluation by FreSHtracer after tumor cells treated with TISN for 0h and 4h. The scale bar is 20μm.*


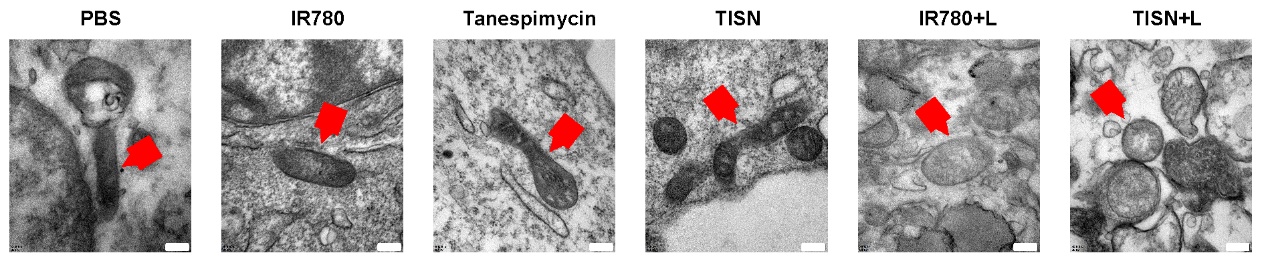


***Figure S10****.* *TEM images of mitochondria in tumor cells treated with PBS, IR780, Tanespimycin, TISN, IR780+L, and TISN+L. The red arrows indicated the mitochondria structure after different treatments. The scale bar is 200nm.*

***
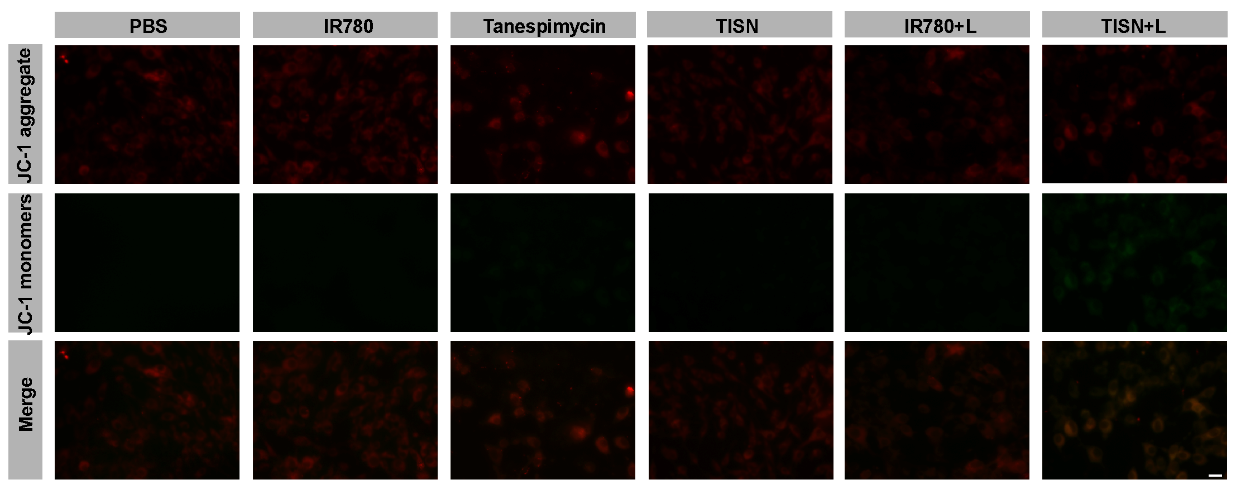
***

***Figure S11****. Mitochondria potential evaluation by JC-1 after tumor cells treated with PBS, IR780, Tanespimycin, TISN, IR780+L, and TISN+L. Red signals (JC-1 aggregates) suggested a normal polarized mitochondrial membrane. Green signals (JC-1 monomers) suggested an abnormal depolarized mitochondrial membrane. The scale bar is 20μm.*

*
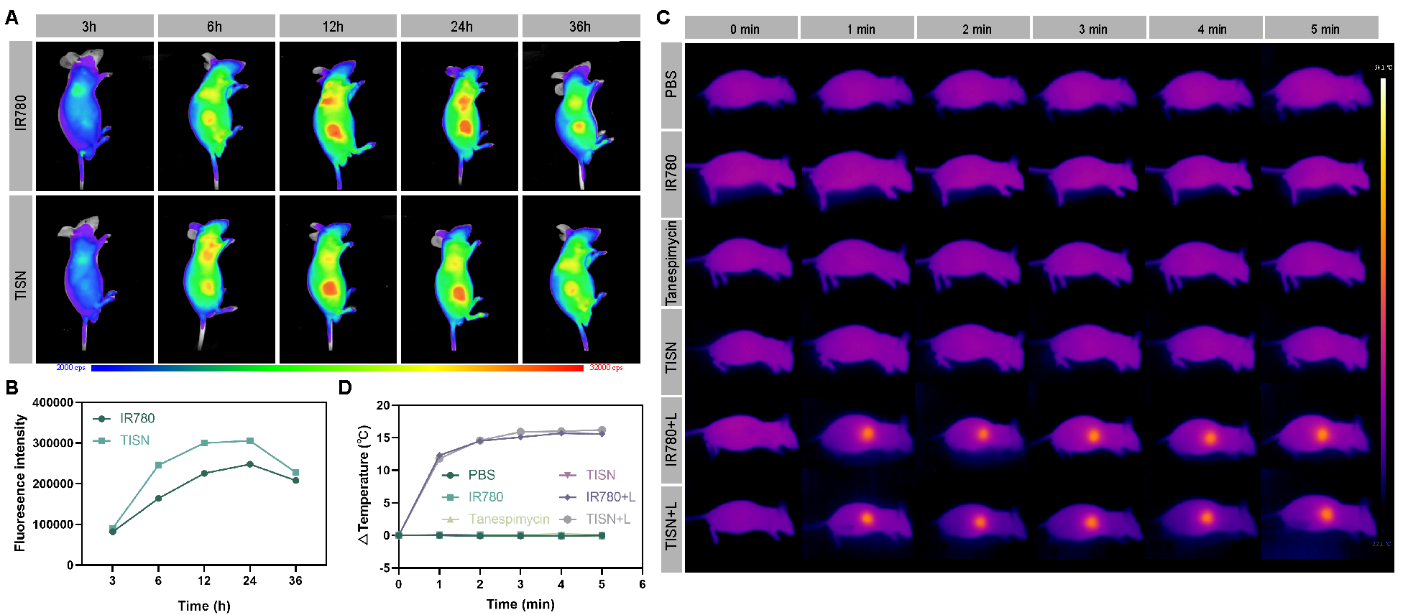
*

***Figure S12.*** *The biodistribution of TISN in vivo. A) The fluorescence images of TISN and IR780 (200μL, 100μg/mL IR780) in the tumor-bearing mice after tail vein injection. B) The quantitative analysis result of Figure 5A. C) The photothermal images of tumor-bearing mice under different treatments after tail vein injection. (1 W/cm^2^). D) The quantitative analysis result of Figure 5C.*

**
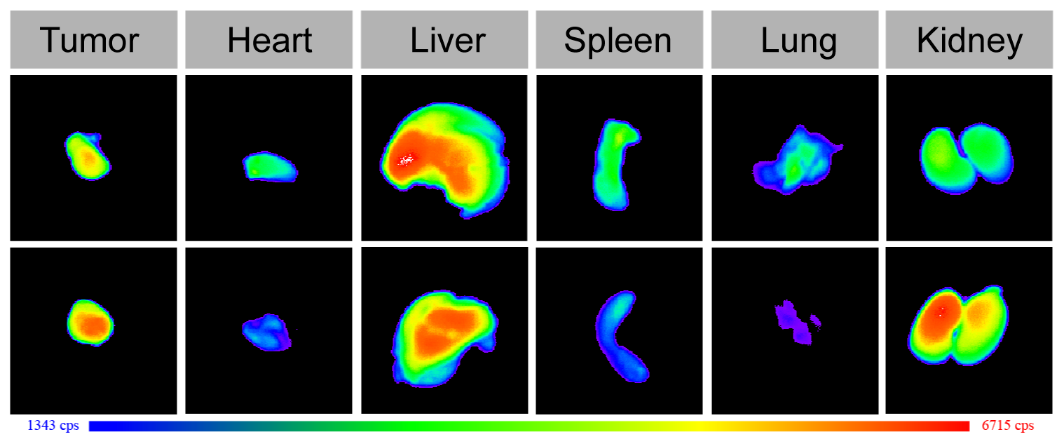
**

­­***Figure S13****. Biodistribution of TISN (200uL, 100ug/mL IR780, tail vein injection). Ex* *vivo NIR images of major organs and tumors at 24h post intravenous injection.*

*
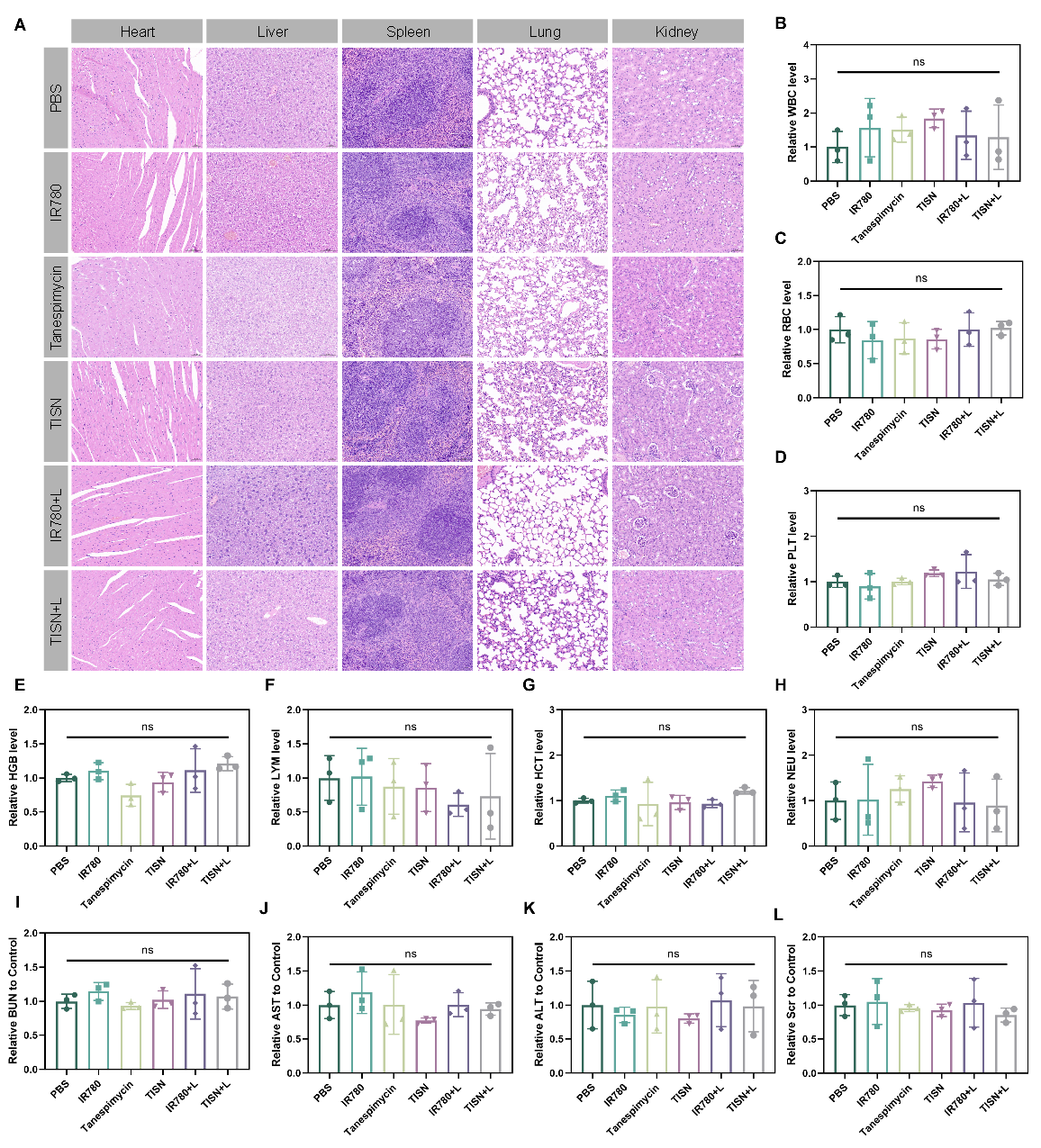
*

***Figure S14.*** *Potential long-term in vivo biosafety analysis of TISN (200μL, 100μg/mL IR780/Tane, tail vein injection). A) H&E staining images of major organs of the AGS-bearing mice in the treatment. The scale bar is 200μm. B-H) Hematology assay (WBC: white blood cell; LYM: lymphocytes; RBC: red blood cell; PLT: platelets;* *NEU: neutrophils; HGB: hemoglobin). I-L) Serum biochemical assay (BUN: blood urea nitrogen; ALT: alanine aminotransferase; AST, aspartate aminotransferase; Scr: serum creatinine). The scale bar is 100μm.*
